# Dataset on the proteomic response during ferroptosis induction via tamoxifen induced GPX4 KO in mouse embryonic fibroblasts

**DOI:** 10.1016/j.dib.2023.109170

**Published:** 2023-04-20

**Authors:** Alexey M. Nesterenko, Dmitry A. Korzhenevskii, Vasilisa M. Tereshchuk, Olga M. Kudryashova, Vsevolod V. Belousov, Arina G. Shokhina

**Affiliations:** a Federal Center of Brain Research and Neurotechnologies, Federal Medical Biological Agency, Moscow 117997, Russia; b Shemyakin-Ovchinnikov Institute of Bioorganic Chemistry, Russian Academy of Science, Moscow 117997, Russia; c Pirogov Russian National Research Medical University, Moscow 117997, Russia

**Keywords:** Ferroptosis, Proteomics, Glutathione peroxidase-4, Mouse embryonic fibroblasts, Pfa1, Cell death

## Abstract

Ferroptosis is a type of programmed cell death distinct from apoptosis and necroptosis that plays an essential role in pathophysiological conditions such as neurodegenerative diseases and tumorigenesis. Massive lipid oxidation in an iron-dependent manner is a hallmark of ferroptosis.This modality of cell death is also characterized by perturbation of several metabolic pathways, predominantly fatty acid metabolism, thiol metabolism, iron homeostasis and the mevalonate pathway.

We aimed to acquire data from different timepoints of ferroptotic death in order to get information about the primary and delayed phases of the ferroptotic response. For this purpose, we used model Pfa1 cells, which are 4-OH-TAM-inducible *Gpx4^−/−^* mouse immortalized fibroblasts [1]. GPX4 is one of the main intracellular ferroptosis regulators and inhibiting it is a classic approach to induce ferroptosis. Measuring protein fold changes at different ferroptotic stages and in nontreated Pfa1 cells could give useful information on the activation of genes involved in ferroptosis and non-genomic protein regulation during ferroptotic progression. Bottom-up proteomic data were acquired from samples obtained 24 and 48 hours after genetic induction of ferroptosis. Chromato-mass spectra were registered in DDA mode and are suitable for further label-free quantification. These data might be a valuable proteome basis for further investigation of ferroptosis and complement other available omics.

**Specifications Table**

**Table:** 

Subject	Omics: Proteomics
Specific subject area	Proteomics of mouse embryonic fibroblasts with tamoxifen inducible deletion of *Gpx4*, referred to as Pfa1 cells, during genetically induced ferroptosis
Type of data	Proteomic data Table
How the data were acquired	DDA-mode proteomics on a HPLC-ESI-MS instrument with an Orbitrap mass-analyzer. Software: MSFragger, peptideProphet, proteinProphet, IonQuant.
Data format	Raw: Thermo RAW files Analyzed: Processed spectra (mzML), Identifications (mzIdentML), Quantities (TSV).
Description of data collection	This dataset represents proteomic analysis on mouse embryonic fibroblasts (MEF) and mouse embryonic fibroblasts with tamoxifen (Tam) inducible deletion of *Gpx4* (referred to as Pfa1 cells). To induce ferroptosis Pfa1 cells were treated with 1 µM Tam. Samples were collected before treatment (0 hour), and at 24 and 48 hours after treatment in 3-4 biological replicates for each time point.
Data source location	Institution: Federal Center of Brain Research and Neurotechnologies City/Town/Region: Moscow, Russia Country: Russia
Data accessibility	Repository name: PRIDE Data identification number: PXD040094 Project Webpage: http://www.ebi.ac.uk/pride/archive/projects/PXD040094 FTP Download: ftp://ftp.pride.ebi.ac.uk/pride/data/archive/2023/04/PXD040094

## Value of the Data

• Pfa1 mouse fibroblasts, a tamoxifen-inducible glutathione peroxidase-4 (GPX4) knockout, is a model cell line that is often used to study the molecular basis of ferroptosis [1]. GPX4 protects lipids from peroxidation, and its knockout results in ferroptotic cell death.
  This is a unique proteome dataset that provides the dynamics of protein levels at time points during ferroptosis. To our knowledge, this is the first proteome data made on a cell line with the 4-OH-TAM-inducible *Gpx4^−/−^* ferroptosis model. There are only a few other proteome studies using different models.
• These data are useful to all researchers who would like to understand the role of ferroptosis at the proteome level and make comparisons with other commonly available omics data. This could provide additional insights into new findings made on other omics.
• Our proteomic profiles may provide new information at the protein level and help in seeking biomarkers that play important roles in ferroptosis in therapy-resistant cancers and degenerative disease pathogeneses.
• These data might be a valuable proteome reference for further investigation of ferroptosis in inducible ferroptosis models.

## 1 Objective

Ferroptosis is a unique type of non-apoptotic programmed cell death with specific features, one of which is the accumulation of lipid peroxides [2]. The key ferroptosis regulatory proteins glutathione peroxidase-4 (GPX4), ferroptosis suppressor protein 1 (FSP1), and long-chain-fatty-acid-CoA ligase (ACSL4), are functionally and topologically linked to intracellular membranes [3], [4], [5]. Therefore, for a long time, the focus has been on lipidomic profiling of model systems undergoing ferroptotic death. However, analysis of proteomic changes may reveal previously undiscovered pathways and lead to the formulation of new hypotheses about the physiological role of ferroptosis, its intracellular initiation sites, ferroptosis-sensitive intracellular compartments, etc.

We used model Pfa1 cells, which are 4-OH-TAM-inducible *Gpx4^−/−^* immortalized mouse fibroblasts [1], to trigger the ferroptosis via *Gpx4* deletion. Bottom-up proteomic data were acquired from samples obtained 24 and 48 hours after genetic induction of ferroptosis. For better usability, we used untreated Pfa1 cells and wild-type mouse embryonic fibroblasts (MEF) as a control. Chromato-mass spectra were registered in DDA mode and are suitable for further label-free quantification.

## 2 Data Description

**Fig. 1:**
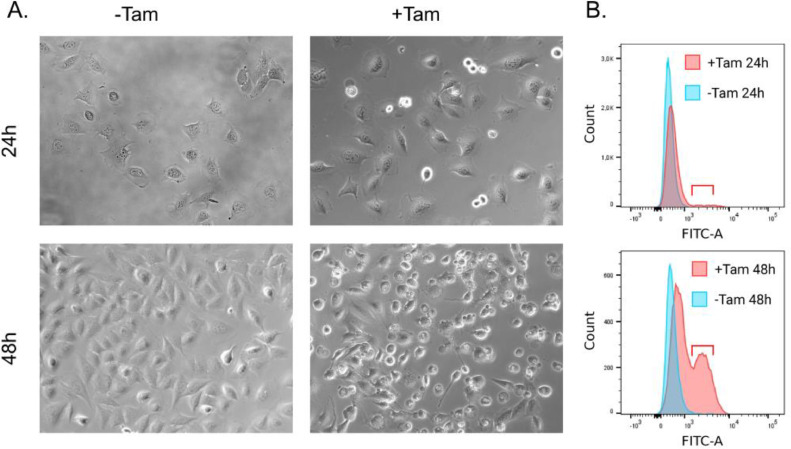
Pfa-1 cells after ferroptosis induction at two time-points for which we report proteomic data. (A) Images of Pfa1 cells treated with or without Tamoxifen, 24h and 48h after treatment. (B) Lipid peroxidation rate evaluated with BODIPY 581/591 C11 probe in Pfa1 cells treated with or without Tamoxifen, 24h and 48h after treatment. The fraction of cells accumulating oxidized lipids is marked with the braket.

In the present study, we report proteome data of mouse embryonic fibroblasts cell lines with tamoxifen-induced GPX4 KO (Pfa1 line). The Pfa1 cell-based model of tamoxifen-dependent GPX4 knockout is a valuable and well characterised tool for ferroptosis research. In this model, ferroptosis develops within 72 hours of induction, and the majority of the population dies. An indirect method to evaluate the development of ferroptosis is based on the ability of the fluorescent probe BODIPY C11 to emit light at 510 nm in oxidized cells. We induced ferroptosis and collected samples at two timepoints: 24h and 48h after tamoxifen addition (Fig. 1A, B). Samples were not collected at 72h as by this point most of the cells are already dead. The early response to ferroptosis induction was evaluated at 24h, when the proportion of cells accumulating oxidized lipids is relatively low according to the BODIPY C11 signal (Fig. 1B, upper, red bracket). 48h after ferroptosis induction, when lipid peroxidation products were detected in a significant proportion of the cell population, the effects of moderate ferroptosis were assessed (Fig. 1B, lower).

For control, we used Pfa1 cells without tamoxifen (referred to as 0h). As an alternative control, we used an MEF cell line provided by Dr. M.Conrad. Additionally, we provide raw data for HEK293 cells as an independent quality control.

**Fig. 2:**
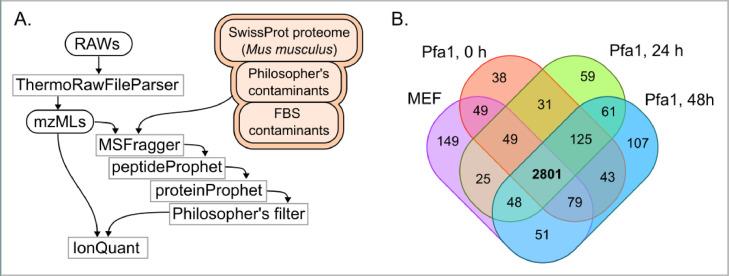
Dataflow pipeline and identification counts in proteomic data. (A) Dataflow diagram starting from Thermo raw files and ending at the IonQuant report table of protein intensities. Databases used are highlighted in orange. (B) A Venn diagram of proteins confidently identified in 4 experimental conditions.

Three biological replicates were prepared for proteomics, and two technical replicates were done for every sample: all these LC-MS2 spectra were uploaded in original Thermo™ RAW format. For analysis, we used the label free quantification (LFQ) protocol carefully described in Methods (Fig. 2A). The LFQ report table in IonQuant format is provided along with raw spectra. Technical replicates are merged at the level of post-search analysis, so we provide one quantity set for one sample. Identified protein counts (criterion: 3 unique PSMs in at least 2 of 3 samples) for different experimental conditions are presented as a Venn diagram (Fig. 2B).

**Fig. 3:**
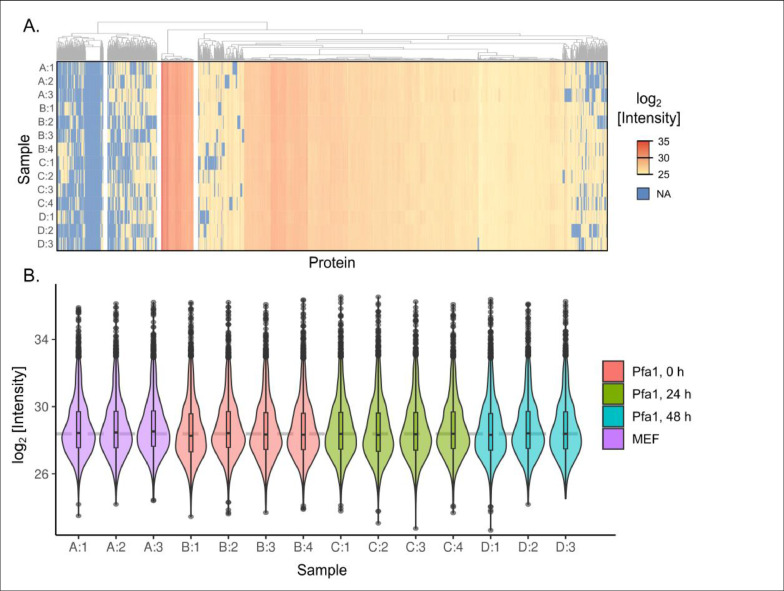
The quality of label-free quantification data. (A) Heatmap of IonQuant razor intensities, missing values are highlighted in blue. (B) Distributions of log2-intensity values of every sample (violin plot). Each of the conditions is represented by color and shortened as A/B/C/D, replicates are referred to by number.

To visualize the quality of the whole dataset, we built a heatmap including missing values (MV) and clusterized genes (Fig. 3A). This heatmap shows: (1) a large group that doesn't contain MVs, (2) a group with moderate amounts of MVs, which could be MNAR-imputed, and (3) a useless group of the dataset with a large amount of MVs, which cannot be imputed. To trust LFQ results, it is important to have very homogeneous MS runs. The distribution of non-normalized log MaxLFQ quantities in all samples demonstrates good inter-sample consistency (Fig. 3B).

We have deposited all the data obtained in the public repository PRIDE (PXD040094). The data includes LC-MS/MS spectra in both RAW and mzML formats, identification information in mzID and PROT.XML formats, and quantification information provided by IonQuant (TSV files). Figs. 3A and 3B demonstrate that our data are of high quality and can be further used for statistical analysis and clustering. The presence of accurate controls (MEF, 0h, and even HEK293) allows data to be obtained in a similar way for direct comparison of protein relative amount changes (for future research).

## 3 Experimental Design, Materials and Methods

### 3.1 Cell Culture

MEF (mouse embryonic fibroblasts) and Pfa1 cells which are 4-OH-TAM-inducible *Gpx4^−/−^* mouse immortalized fibroblasts were gifted from Dr. Marcus Conrad (Munich, Germany). Pfa1 cells were cultured in RPMI-1640 medium (PanEko) containing glucose (2 g/L), fetal bovine serum (10%), Gibco GlutaMAX Supplement (2 mM) and pen/strep (1%) at 37 °C with 5% CO2 in a humidified incubator. Pfa1 cells (300000) were seeded on Corning 100 mm tissue-culture treated culture dishes (#CLS430167) and incubated overnight. On the next day cells were treated with or without 1 µM (Z)-4-Hydroxytamoxifen (H7904, Sigma-Aldrich).

### 3.2 Lipid Peroxidation Evaluation with BODIPY 581/591 C11 Probe

Pfa1 cells (115000) were seeded on 60 mm tissue-culture treated dishes and incubated overnight. On the next day cells were treated with or without 1 µM (Z)-4-Hydroxytamoxifen (H7904, Sigma-Aldrich). 23 and 47 hours later, cells were treated with 1.5 µM BODIPY 581/591 C11 (Thermo Fisher) for 1 hour at 37 °C. Then cells were trypsinized, resuspended in PBS (500 µL) and analyzed with a BD FACSCanto Flow Cytometer (488 laser for excitation, 530/30 band-pass filter). At least 30000 cells were analyzed per sample. Data analysis was performed with FlowJo software.

### 3.3 Protein Extraction, Purification, and Processing

The original trifluoroethanol-based protocol [6] was modified for our setup. Cells (about 1.10^6^) were washed three times with PBS, then treated with 80% cold methanol, scraped from the dishes and incubated for 1 hour at -80 °C. The protein-containing pellet was collected by centrifugation. The pellet was washed with cold acetone, centrifuged again and dried in air. The proteins were redissolved in 120 µl of 50% trifluoroethanol solution in 50 mM NH_4_HCO_3_ buffer pH 8.0 by ultrasonication, then treated sequentially with 5 mM TCEP (1 hour, 50 °C) and 15 mM iodoacetamide (1 hour, RT). Then the samples were diluted 4 times with 50 mM NH_4_HCO_3_ buffer pH 8.0, 2 µg of trypsin/LysC mix (Promega, USA) added to each, and incubated overnight at 37 °C. Enzymatic cleavage was stopped by adding formic acid up to 1%, samples were centrifuged and the supernatants dried on a vacuum centrifugation device (Labconco CentriVap) at 45 °C. Peptide content in each sample was measured using a BCA Protein Assay kit (Merck Millipore, USA).

### 3.4 HPLC-MS^2^ Analysis

Shutgun proteomic analysis was performed using a method described earlier by the employees of the core facility [7] with minor modifications using an Ultimate 3000 RSLCnano HPLC system (Thermo Scientific, USA) and Q-Exactive HF-X mass spectrometer (Thermo Scientific, USA).The peptide mixture (1 µg) was loaded onto an Acclaim µ-Precolumn enrichment column (0.5 mm × 3 mm, particle size 5 µm, Thermo Scientific) at 10 µl/min flow for 1.5 min in isocratic mode using buffer C as the mobile phase (2% Acetonitrile, 0.1% formic acid in deionized water). Then the peptides were separated on an Acclaim Pepmap® C18 HPLC column (75 µm × 150 mm, particle size 2 µm) (Thermo Scientific, USA) in gradient elution mode (mobile phase A: 0.1% formic acid, B: 80% acetonitrile, 0.1% aqueous formic acid solution, flow rate: 0.4 µl/min). The following 110-min gradient was used: 2% B (1 min), 2 → 35% B (94 min), 35 → 99% B (4 min), 99% B (5min), 99 → 2% B (2 min), 2% B (5 min).

The mass-spectrometer was operated in positive ionization mode using a NESI source (Thermo Scientific, USA). The parameters for the MS/MS-analysis were as follows: 2.1 kV emitter voltage, 240 °C capillary temperature. Panoramic scanning was performed in the mass range 390 – 1500 m/z at 120000 resolution. For the tandem scan, the resolution was 15000 and the mass range was from 100 m/z to the automatically selected upper limit. Isolation of precursor ions was performed in the ± 1 Da window. The top 20 precursors in a charge range (+2 – +4) were isolated in tandem mode, the intensity cutoff limit for precursor selection was set to 40000 arbitrary units, and the normalized collision energy (NCE) - to 29 units. The maximum accumulation times for precursor and fragment ions were 50 ms and 100 ms, correspondingly. The AGC values were 1·10^6^ and 2·10^5^, respectively for former and later ions. Processed precursor ions were dynamically excluded from MS/MS analysis for 20 s.

### 3.5 Label Free Quantification Protocol

Thermo RAW files were first converted into mzML format using ThermoRawFileParser [8]. Next, a closed database search was performed using MSFragger 3.5 [9] with a true precursor tolerance of 12 ppm and fragment mass tolerance of 50 mDa. 3 missed cleavages were allowed, precursor ions with z=2-4 were considered. Three variable modifications were used: methionine oxidation, N-terminal acetylation, and cysteine carbamidomethylation. We used a standard mouse SwissProt proteome (UP000000589) augmented with standard contaminants of the Philosopher package [10] and with FBS contaminants measured previously in our lab on FBS samples using the same instrument.

Initial target-decoy analysis was performed with peptideProphet [11] fed with every group of search output files corresponding to different technical replicates of one sample. Next-level statistical refinement was performed using proteinProphet [12] on all peptideProphet output files. Finally, after refinement result filtration we performed label free quantification using IonQuant 1.8 with the match-between-run option turned on [13].

The described protocol is schematically presented in Fig. 2A.

## Ethics Statements

The manuscript complies with the publication's ethics standards. Only immortalized mouse cell lines were used in the study.

## CRediT authorship contribution statement

**Alexey M. Nesterenko:** Formal analysis, Data curation, Visualization, Writing – review & editing. **Dmitry A. Korzhenevskii:** Investigation, Writing – original draft. **Vasilisa M. Tereshchuk:** Formal analysis, Visualization, Writing – original draft. **Olga M. Kudryashova:** Formal analysis, Data curation, Writing – review & editing. **Vsevolod V. Belousov:** Supervision. **Arina G. Shokhina:** Conceptualization, Resources, Writing – review & editing, Project administration, Funding acquisition.

## Declaration of Competing Interest

The authors declare that they have no known competing financial interests or personal relationships that could have influenced the work reported in this paper.

## Data Availability

Proteomic response on ferroptosis induction via GPX4 KO in mouse embryonic fibroblasts (Original data) (PRIDE).
